# Differential activation of TCA cycle activity and PDH flux by dichloroacetate in skeletal muscle measured by hyperpolarized [2-^13^C,3-^2^H_3_]pyruvate

**DOI:** 10.1016/j.isci.2026.117325

**Published:** 2026-08-27

**Authors:** Sung-Han Lin, Andrew Cho, Mai T. Huynh, Zohreh Erfani, Blanka Kucejova, Sasanka Wathukara Dewage, Thomas Jue, Xiaorong Fu, Zoltán Kovács, Shawn C. Burgess, Jae Mo Park

**Affiliations:** 1 Advanced Imaging Research Center, University of Texas Southwestern Medical Center, Dallas, TX, USA; 2 Department of Radiology, University of Texas Southwestern Medical Center, Dallas, TX, USA; 3 Center for Human Nutrition, University of Texas Southwestern Medical Center, Dallas, TX, USA; 4 Department of Biochemistry and Molecular Medicine, University of California, Davis, Davis, CA, USA; 5 Department of Internal Medicine, University of Texas Southwestern Medical Center, Dallas, TX, USA; 6 Department of Pharmacology, University of Texas Southwestern Medical Center, Dallas, TX, USA; 7 Department of Biomedical Engineering, University of Texas Southwestern Medical Center, Dallas, TX, USA; 8 Charles and Jane Pak Center for Mineral Metabolism and Clinical Research, University of Texas Southwestern Medical Center, Dallas, TX 75390, USA

**Keywords:** skeletal muscle, oxidative phosphorylation, hyperpolarized, pyruvate, pyruvate dehydrogenase, dichloroacetate, acetyl-L-carnitine, TCA cycle

## Abstract

Despite the central role of skeletal muscle bioenergetics in whole-body metabolic health, assessing mitochondrial oxidative phosphorylation and tricarboxylic acid (TCA) cycle activity *in vivo* remains a major challenge. While hyperpolarized [1-^13^C]pyruvate has been used to probe pyruvate dehydrogenase (PDH) flux to approximate TCA cycle activity, this approach relies on the unreliable assumption that PDH and TCA cycle fluxes are tightly coupled. Here, we demonstrate that hyperpolarized [2-^13^C,3-^2^H_3_]pyruvate can track label-incorporation into TCA cycle-derived glutamate in rat skeletal muscle. Following intravenous dichloroacetate administration, we observed a greater increase in hyperpolarized [1-^13^C]acetyl-L-carnitine relative to [5-^13^C]glutamate, suggesting disproportionately increased PDH flux relative to TCA cycle flux. A similar trend was also observed in *ex vivo* GC-MS analysis of skeletal muscle tissue collected from rats injected with [U-^13^C_3_]pyruvate. Together, these findings highlight the complex interplay between PDH and TCA cycle fluxes and establish hyperpolarized [2-^13^C,3-^2^H_3_]pyruvate as a robust agent for assessing mitochondrial metabolism in skeletal muscle.

## Highlights

• *In vivo* assessment of divergent fates of PDH-derived acetyl-CoA in skeletal muscle
• PDH flux can increase disproportionately relative to TCA cycle flux *in vivo*
• Acetyl-L-carnitine buffers excess PDH-derived acetyl-CoA in skeletal muscle

## Graphical abstract

**Figure:**
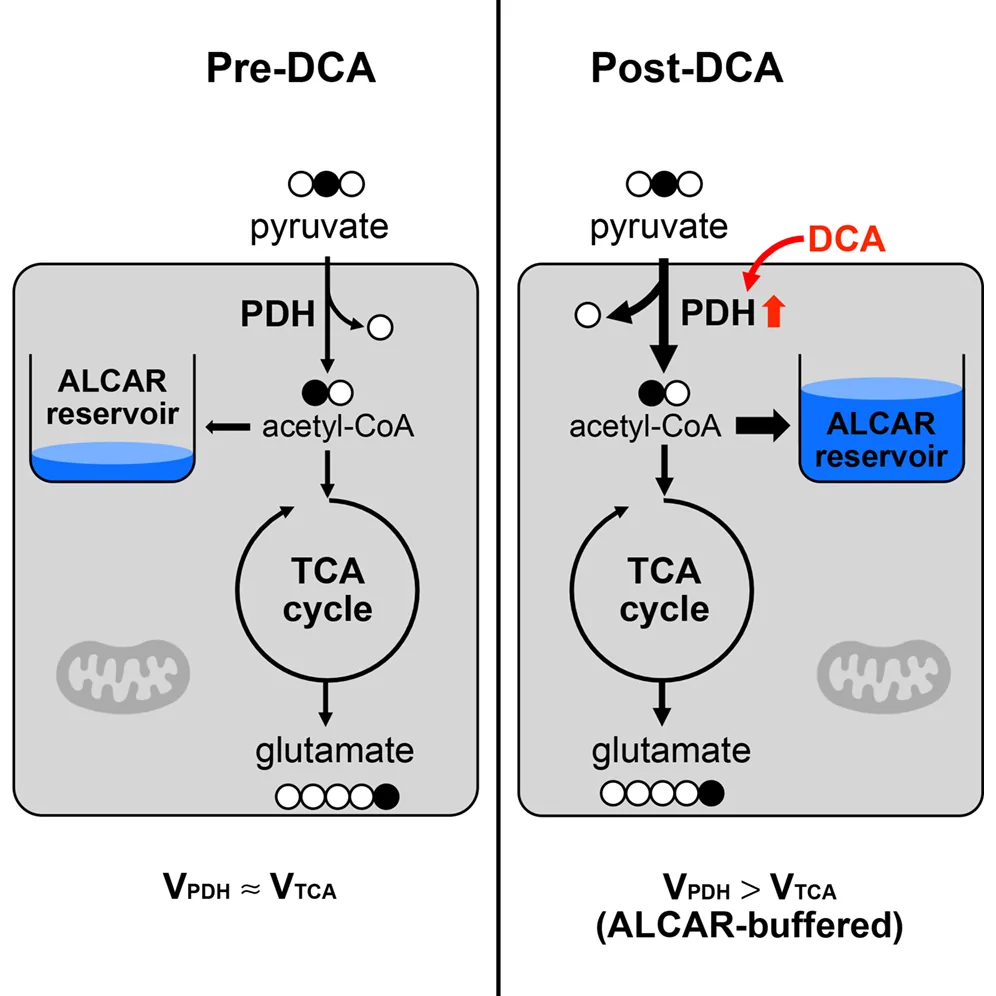

## Teaser

Molecular biology; Cell biology

## Introduction

Skeletal muscle is a metabolically active tissue which relies on mitochondrial oxidative phosphorylation (OXPHOS) and the tricarboxylic acid (TCA) cycle to sustain energy production. Together, these pathways supply the majority of adenosine triphosphate (ATP) required for prolonged contraction under aerobic conditions. During sustained or repeated muscle activity, the TCA cycle and OXPHOS become the dominant mechanisms for ATP generation, efficiently oxidizing carbohydrates and fatty acids.^1^ Impaired OXPHOS and mitochondrial function contribute to a variety of metabolic diseases, such as insulin resistance and type 2 diabetes, in which reduced oxidative capacity disrupts substrate utilization and decreases metabolic flexibility.^2^ Investigating how skeletal muscle regulates OXPHOS and TCA cycle activity is therefore essential to improve our understanding of skeletal muscle physiology, which can be leveraged toward the discovery of novel therapeutic strategies. However, real-time assessment of OXPHOS and TCA cycle activity *in vivo* remains a major challenge, limiting mechanistic insight into skeletal muscle metabolism in both health and disease.

Skeletal muscle is a highly adaptive tissue which rapidly modulates its energetic pathways in response to external stimuli and fluctuating energy demand, such as abrupt increases in exercise load or alterations in the tissue microenvironment. Understanding this dynamic metabolic landscape, and how it is perturbed under pathological conditions, requires *in vivo* approaches that preserve physiological context. Existing *in vivo* methods such as ^31^P magnetic resonance spectroscopy (MRS) and oxygen respirometry provide bulk measurements of phosphate metabolite concentrations and whole-body energetics, respectively, but these approaches do not resolve substrate-specific fluxes or downstream TCA cycle activity within skeletal muscle mitochondria.

To circumvent these limitations, hyperpolarized (HP) ^13^C MRS has emerged as a powerful noninvasive tool to probe real-time metabolic fluxes *in vivo*. The most widely studied HP probe to date is [1-^13^C]pyruvate, which can simultaneously interrogate flux through several major cytosolic and mitochondrial enzymes.^3^ Among these, pyruvate dehydrogenase (PDH) is of particular importance in skeletal muscle physiology since it regulates the entry of pyruvate into the TCA cycle. PDH flux can be directly interrogated with HP [1-^13^C]pyruvate by monitoring its conversion to [^13^C]bicarbonate, and elevated [^13^C]bicarbonate production has been observed during aerobic plantar flexion exercise in human calf muscle.^4^

However, a major drawback of [1-^13^C]pyruvate is that PDH-mediated oxidation sequesters the ^13^C label into the bicarbonate pool and thereby prevents ^13^C-labeling of acetyl-CoA and downstream TCA cycle metabolites. Nevertheless, because HP [1-^13^C]pyruvate has several technical advantages compared to other HP tracers, PDH-mediated conversion of [1-^13^C]pyruvate to [^13^C]bicarbonate is regularly used as a surrogate for mitochondrial OXPHOS. Likewise, PDH flux (V_PDH_) is frequently equated with TCA cycle flux (V_TCA_) in metabolism models.^5^ However, these relationships have not been validated since [1-^13^C]pyruvate cannot directly assess metabolism downstream of PDH. Moreover, V_PDH_ likely overestimates V_TCA_ and OXPHOS since the acetyl-CoA pool is dynamically coupled to the acetyl-L-carnitine (ALCAR) pool,^6^^,^^7^ which buffers mitochondrial acetyl availability.^8^

In contrast to [1-^13^C]pyruvate, PDH-mediated oxidation of [2-^13^C]pyruvate generates [1-^13^C]acetyl-CoA which subsequently enters the TCA cycle and facilitates ^13^C-incorporation into various downstream metabolites such as citrate and glutamate. Given the ability of this tracer to directly label TCA cycle metabolites, prior studies have attempted to utilize HP [2-^13^C]pyruvate to approximate V_TCA_.^7^^,^^9^^,^^10^ Specifically, conversion of [2-^13^C]pyruvate to [5-^13^C]glutamate has emerged as a reliable readout of TCA cycle activity.^9^^,^^11^^,^^12^ However, [2-^13^C]pyruvate suffers from a substantially shorter spin-lattice relaxation time (*T*_*1*_ = ∼39 s at 3T) compared to [1-^13^C]pyruvate (∼67 s at 3T) which results in decreased *in vivo* signal-to-noise ratio. This limitation arises from enhanced dipolar and scalar interactions with neighboring protons, accelerating signal decay and decreasing sensitivity for mitochondrial products.^13^ As a result, conversion of [2-^13^C]pyruvate to [5-^13^C]glutamate is not reliably detected in skeletal muscle under conditions associated with low PDH flux, such as rest.^10^^,^^11^ To address this limitation, we previously reported a method to prolong the pyruvate carbon-2 *T*_*1*_ via selective deuteration of the methyl group ([2-^13^C,3-^2^H_3_]pyruvate) and performing hyperpolarized substrate dissolution with deuterium oxide (D_2_O), thereby facilitating reliable *in vivo* measurement of [5-^13^C]glutamate and V_TCA_.^12^

In this study, we build upon our prior work and evaluate the feasibility of HP [2-^13^C,3-^2^H_3_]pyruvate to probe TCA cycle activity in skeletal muscle. We interrogated skeletal muscle metabolism with HP [2-^13^C,3-^2^H_3_]pyruvate MRS before and after intravenous administration of dichloroacetate (DCA), which stimulates V_PDH_ via inhibition of pyruvate dehydrogenase kinase (PDK).^14^^,^^15^ In parallel, *ex vivo* isotopomer analysis was performed to measure the concentration and fractional enrichment of pyruvate-derived metabolites to validate the findings from our *in vivo* HP MRS experiments. Collectively, our results demonstrate that HP [2-^13^C,3-^2^H_3_]pyruvate is a robust probe for interrogating mitochondrial metabolism in skeletal muscle under resting and activated conditions.

## Results

### Assessment of DCA effects on pyruvate metabolism in skeletal muscle using HP pyruvate

**Figure 1:**
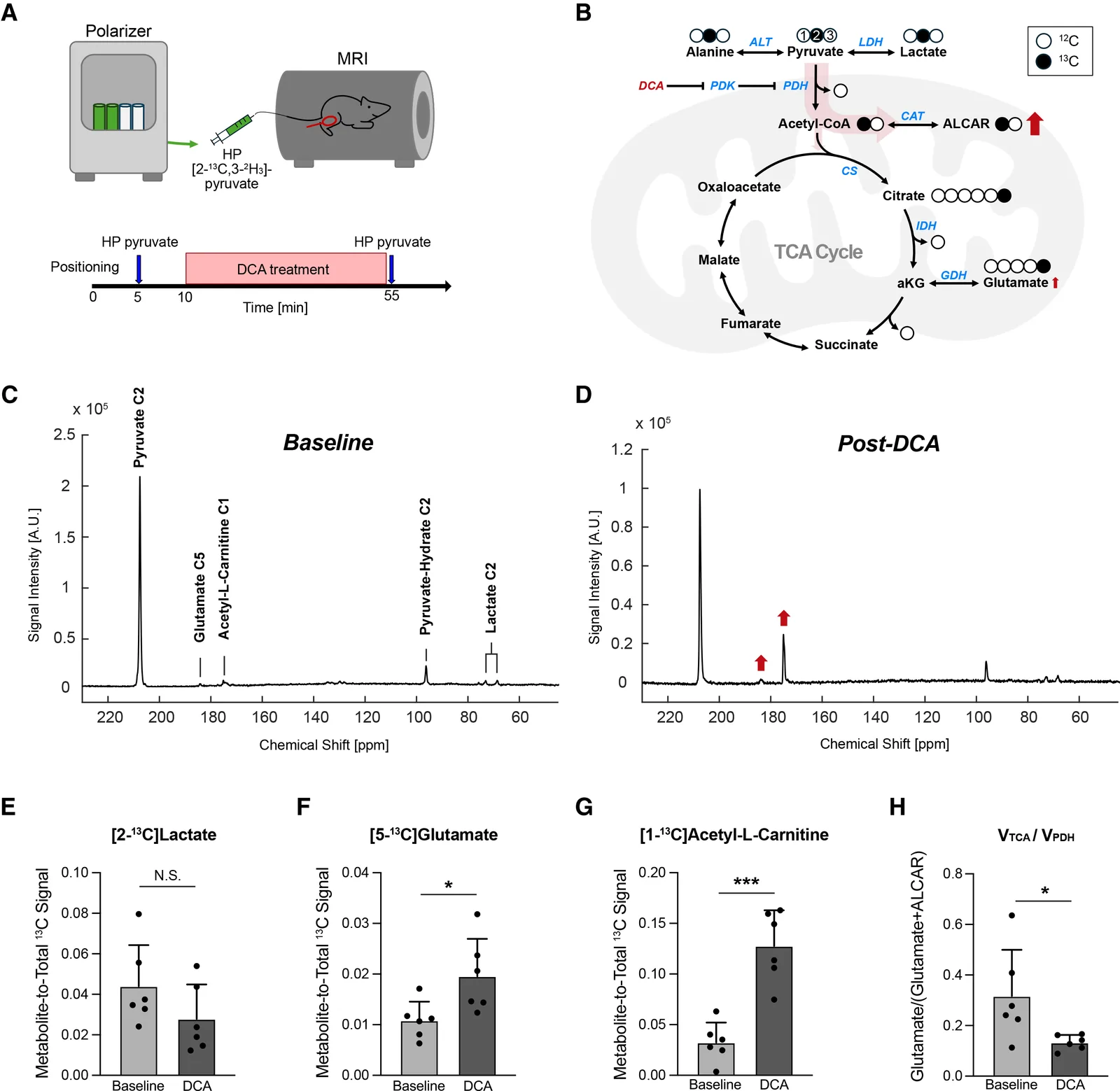
DCA-induced pyruvate metabolism in skeletal muscle measured *in vivo* using ^13^C MRS with HP [2-^13^C,3-^2^H_3_]pyruvate (A) *In vivo* experimental design using a DNP polarizer, a ^13^C surface coil, and a 3T MRI scanner. DCA was injected prior to the second injection of HP pyruvate. (B) Schematic of the metabolic fates of [2-^13^C,3-^2^H_3_]pyruvate, demonstrating ^13^C-incorporation into PDH. Time-averaged ^13^C spectra (0-90s) from representative rat skeletal muscle after injection of 80 mM HP [2-^13^C,3-^2^H_3_]pyruvate at (C) baseline and (D) post-DCA. (E) No DCA-induced change was detected in [2-^13^C]lactate/TC. (F) [5-^13^C]Glutamate/TC and (G) [1-^13^C]acetyl-L-carnitine/TC were elevated following DCA treatment. (H) The ratio of [5-13C]glutamate to ([1-13C]acetyl-L-carnitine + [5-13C]glutamate) decreased after DCA treatment, implying decreased V_TCA_/V_PDH_. aKG, alpha-ketoglutarate; CAT, carnitine acetyltransferase; CS, citrate synthase; DCA, dichloroacetate; GDH, glutamate dehydrogenase; LDH, lactate dehydrogenase; PDH, pyruvate dehydrogenase; PDK, pyruvate dehydrogenase kinase; TC, total HP ^13^C signal; TCA, tricarboxylic acid. Data are represented as mean ± SD. Paired *t* tests (two-tailed, α = 0.05). ∗*p* < 0.05, ∗∗*p* < 0.01, ∗∗∗*p* < 0.001.

To evaluate *in vivo* pyruvate metabolism with HP ^13^C MRS, six healthy male Sprague-Dawley rats were injected with an intravenous bolus of HP [2-^13^C,3-^2^H_3_]pyruvate at baseline and 45 min after intravenous injection of DCA (Figure 1). By extending the *T*_*1*_ relaxation time of the pyruvate carbonyl carbon via deuteration of the methyl hydrogens and performing dissolution in D_2_O, the *in vivo* signal-to-noise ratio was sufficient for reliable detection of mitochondrial metabolites such as [5-^13^C]glutamate and [1-^13^C]ALCAR across all experimental groups. When normalized to the total HP ^13^C signal (TC), we observed a small and statistically insignificant decrease in [2-^13^C]lactate/TC following DCA treatment from 0.0440 ± 0.0209 to 0.0279 ± 0.0170 (paired two-tailed *t* test, *p* = 0.167). In contrast, [5-^13^C]glutamate/TC increased from 0.0116 ± 0.0030 to 0.0196 ± 0.0146 (*p* = 0.033) and [1-^13^C]ALCAR/TC increased from 0.0330 ± 0.0188 to 0.1277 ± 0.0352 (*p* = 0.0002). The magnitude of this increase was larger for [1-^13^C]ALCAR/TC (418.5 ± 305.1%) compared to [5-^13^C]glutamate/TC (77.9 ± 81.9%). The metabolite ratio of [5-^13^C]glutamate to the sum of [5-^13^C]glutamate and [1-^13^C]ALCAR was also calculated as a marker of the TCA cycle to PDH flux ratio (V_TCA_/V_PDH_). This metric was 45.5 ± 33.1% lower after DCA treatment (0.1421 ± 0.0229) compared to baseline (0.3150 ± 0.1792, *p* = 0.034), suggesting increased PDH activity relative to TCA cycle flux. Alanine and citrate peaks were below the detection limit.

From the time-resolved ^13^C spectra, time courses of HP [2-^13^C]pyruvate, [2-^13^C]lactate, [5-^13^C]glutamate, and [1-^13^C]ALCAR were also acquired (Figure S1). DCA substantially increased [1-^13^C]ALCAR production without altering the time-to-peak of the signal (36.3 ± 4.3 s to 40.7 ± 8.2 s, *p* = 0.561), suggesting that the increase in [1-^13^C]ALCAR signal is attributed to DCA-mediated PDH activation rather than differences in perfusional delivery of pyruvate between groups. Similarly, the time-to-peak for [2-^13^C]lactate was not shifted by DCA (control: 27.0 ± 4.2 s, post-DCA: 26.4 ± 5.4 s, *p* = 0.799). Dynamic changes of [5-^13^C]glutamate could not be reliably quantified due to limited signal sensitivity of time-resolved spectra.

### Validation of HP MRS findings in skeletal muscle using *ex vivo* GC-MS

**Figure 2:**
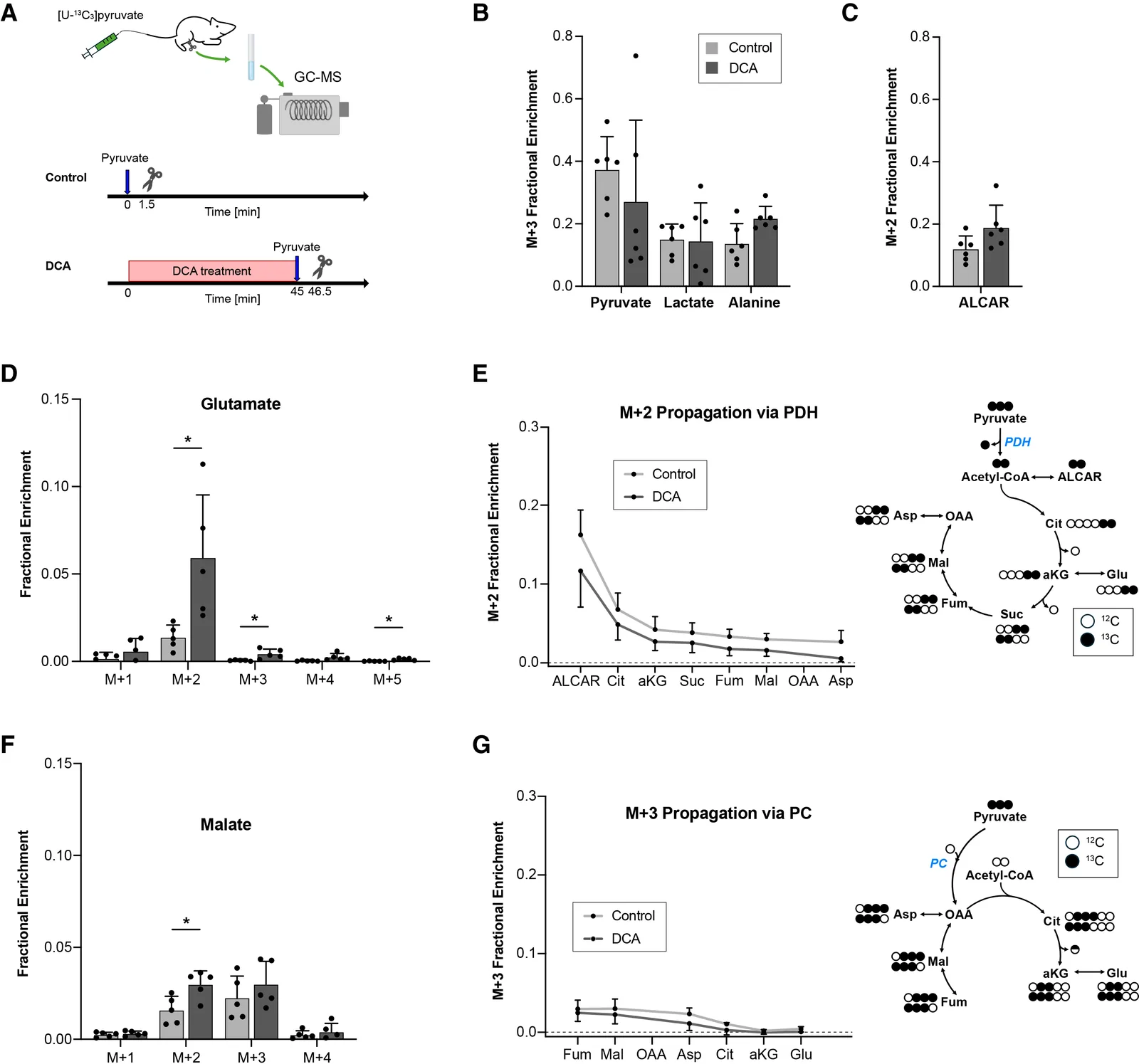
*Ex vivo* isotopologue analysis of skeletal muscle with GC-MS (A) Control and DCA-treated groups were injected with [U-^13^C_3_]pyruvate 90 s prior to tissue collection for GC-MS analysis. (B) Fractional abundance of M+3 pyruvate, lactate, and alanine were comparable between the groups. (C) M+2 ALCAR mildly increased in the DCA-treated group without statistical significance. (D) M+2 glutamate was significantly increased with DCA treatment. (E) PDH-mediated M+2 enrichment propagated further into the TCA cycle in DCA-treated animals compared with controls (*p* < 0.0001). (F) Malate M+2 enrichment was significantly higher in DCA-treated animals. (G) M+3 labeling did not propagate differently between groups and did not extend substantially beyond the four-carbon intermediates, with minimal M+3 detected beyond the four-carbon intermediates. aKG, alpha-ketoglutarate; ALCAR, acetyl-L-carnitine; Asp, aspartate; Cit, citrate; DCA, dichloroacetate; Fum, fumarate; Glu, glutamate; Mal, malate; OAA, oxaloacetate; PC, pyruvate carboxylase; PDH, pyruvate dehydrogenase; Suc, succinate. Data are represented as mean ± SD. Unpaired *t* tests (two-tailed, α = 0.05). ∗*p* < 0.05.

To validate the HP findings, a separate cohort of six healthy male Sprague-Dawley rats were injected with an intravenous bolus of [U-^13^C_3_]pyruvate and the bilateral hamstring muscles were excised 90 s post-injection (Figure 2A). Three of these rats received intravenous DCA 45 min prior to [U-^13^C_3_]pyruvate injection, whereas the remaining three were untreated. Following tissue extraction, fractional enrichment and concentrations of various pyruvate-derived metabolite isotopologues were measured by gas chromatography-mass spectrometry (GC-MS). Pyruvate, lactate, and alanine M+3 enrichments were not significantly different between groups (Figure 2B). ALCAR M+2 fractional enrichment exhibited a small and statistically insignificant increase following DCA treatment (control: 0.1200 ± 0.0416, post-DCA: 0.1890 ± 0.0717, unpaired two-tailed *t* test, *p* = 0.076) (Figure 2C). Meanwhile, the fractional abundance of glutamate M+2 was elevated following DCA treatment (control: 0.0136 ± 0.0063, post-DCA: 0.0560 ± 0.0331, *p* = 0.011), likely corresponding to increased [4,5-^13^C_2_]glutamate production from DCA-mediated PDH activation and elevated TCA cycle flux (Figure 2D).

Since HP signal intensity is proportional to the concentration of labeled metabolite, we also calculated the product of the MS-derived metabolite concentration and fractional enrichment to estimate the total amount of labeled metabolite. Glutamate M+2 concentration was markedly increased by DCA (control: 13.72 ± 6.01 nmol/g; post-DCA: 91.06 ± 51.80 nmol/g; *p* = 0.005; Figure S2). The glutamate M+2 concentration was also normalized to the sum of glutamate M+2 and ALCAR M+2 to approximate V_TCA_/V_PDH_, analogous to the HP-based calculation shown in Figure 1H. Interestingly, this ratio was higher in the DCA-treated group (0.9850 ± 0.0118) compared to controls (0.9264 ± 0.0451, *p* = 0.012), opposite to the HP-derived estimate. However, ALCAR M+2 enrichment closely resembled alanine M+3 enrichment at 90 s, suggesting that transfer of pyruvate-derived carbon into the ALCAR pool occurred rapidly relative to downstream TCA-cycle labeling, complicating interpretation of its apparent rate. In contrast, glutamate M+2 enrichment remained substantially lower than alanine M+3 (control: 10.3 ± 3.4%, post-DCA: 26.7 ± 18.0%), indicating that TCA cycle labeling was far from isotopic steady state. Under these conditions, differences in V_TCA_ are likely captured by differences in glutamate M+2 accumulation whereas differences in V_PDH_ cannot be reliably assessed via ALCAR M+2 enrichment.

Because 90 s of labeling permits only limited TCA cycle turnover, we then examined the propagation of M+2 labeling (derived from [1,2-^13^C_2_]acetyl-CoA) across sequential TCA cycle intermediates as an alternative index of V_TCA_. M+2 enrichment extended further along the TCA cycle in muscle from DCA-treated animals compared with controls (Figure 2E). Assuming sequential progression of label through the cycle, nonlinear regression to an exponential decay model revealed distinct differences in propagation behavior between control and DCA-treated animals (extra sum-of-squares F test, *p* < 0.0001). The estimated propagation constant was K = 1.01 (95% CI 0.58–1.81) for control and K = 1.51 (95% CI 1.16–2.06) for DCA-treated animals, consistent with increased V_TCA_ in the DCA-treated cohort.

A caveat of the propagation constant calculations described above is they rely on the assumption that PDH entry and citrate synthesis are quantitatively captured by M+2 labeling. This assumption would be violated in the setting of significant pyruvate carboxylase (PC) flux, which would generate labeled oxaloacetate that could subsequently condense with labeled acetyl-CoA. To evaluate the contribution of PC toward overall TCA cycle flux, we examined the labeling patterns of the four-carbon TCA cycle intermediates. Given the absence of appreciable glutamate isotopologues other than M+2, the M+3 labeling observed in malate (Figure 2F) and other four-carbon intermediates is consistent with PC-mediated formation of M+3 oxaloacetate, which equilibrates with malate via malate dehydrogenase. In contrast to M+2 labeling, M+3 enrichment did not propagate differently between groups and remained largely confined to four-carbon intermediates, with minimal M+3 detected in citrate and only trace enrichment in glutamate (Figure 2G). These findings are consistent with relatively low PC flux compared with citrate synthase (CS)-mediated entry of acetyl-CoA under these conditions and support the use of M+2 labeling to capture PDH- and CS-derived entry into the TCA cycle. The concentrations of the metabolites are summarized in Figure S2.

## Discussion

HP [2-^13^C]pyruvate has been suggested as a robust HP probe for direct assessment of TCA cycle activity since the ^13^C-enriched carbon is directly incorporated into various TCA cycle metabolites.^7^^,^^9^ This offers a distinct advantage compared to [1-^13^C]pyruvate which cannot access pathways beyond PDH and therefore cannot directly probe V_TCA_. Nonetheless, due to the superior relaxation properties and sensitivity of HP [1-^13^C]pyruvate, PDH-derived [^13^C]bicarbonate is more frequently used as a surrogate for V_TCA_ under the assumption that PDH and TCA cycle activity are directly proportional. To overcome the poor relaxation properties of [2-^13^C]pyruvate, we instead used HP [2-^13^C,3-^2^H_3_]pyruvate and performed dissolution in D_2_O to prolong the *T*_*1*_ relaxation time of the carbonyl carbon and facilitate reliable *in vivo* detection of various mitochondrial metabolites. Notably, we could reliably detect HP [5-^13^C]glutamate in both resting and DCA-treated skeletal muscle following injection with [2-^13^C,3-^2^H_3_]pyruvate, which is a significant improvement over HP [2-^13^C]pyruvate which can only reliably detect HP [5-^13^C]glutamate in DCA-treated muscle.^10^^,^^11^

Using this probe, we also observed that DCA treatment resulted in a disproportionately larger increase in HP [1-^13^C]ALCAR relative to HP [5-^13^C]glutamate, consistent with a mismatch between PDH-driven acetyl production and net TCA cycle turnover (V_PDH_ > V_TCA_). This finding underscores the need for *in vivo* tools that separately interrogate V_PDH_ and V_TCA_ to facilitate accurate assessment of acetyl-CoA generation and its subsequent oxidation.

Altered production of ALCAR from HP [2-^13^C]pyruvate has been reported primarily in cardiac tissue, where pyruvate oxidation via PDH is essential for energy production. The initial observation was made in perfused heart studies by Schroeder et al.,^7^ who identified ALCAR as a reservoir for disposing of excess acetyl-CoA during periods of low energy demand, as well as for replenishing acetyl-CoA in the setting of increased cardiac output. Subsequently, Sonal et al.^9^ demonstrated that *in vivo* ALCAR production in the heart increased in response to DCA-mediated PDH activation, whereas it remained near baseline when cardiac workload was elevated using dobutamine. In contrast, [5-^13^C]glutamate production increased with both DCA and dobutamine, but they did not report relative changes in ALCAR compared to glutamate as we did in our study.

Our observation in resting skeletal muscle is relevant to the “ALCAR overflow pool” hypothesis. If DCA-induced activation of PDH results in a greater increase in HP [1-^13^C]ALCAR relative to HP [5-^13^C]glutamate, the conventional assumption that V_PDH_ contributes solely to V_TCA_ may underestimate total PDH-derived acetyl flux. In this context, DCA-stimulated PDH activity may preferentially increase acetyl-CoA buffering through the ALCAR pool rather than drive proportionate increases in TCA cycle turnover. The approximately 2-fold increase in HP glutamate signal observed in our study is notable given the absence of increased contractile demand; however, it is consistent with prior *in vivo* kinetic analyses of skeletal muscle ^13^C-glutamate enrichment in rats treated with triiodo-*l*-thyronine, which was attributed to activation of mitochondrial uncoupling.^16^ Collectively, these findings support a model in which ALCAR serves as a dynamic buffer for excess PDH-derived acetyl-CoA via the carnitine acetyltransferase (CAT) system^7^ when acetyl-CoA production exceeds oxidative demand.

*Ex vivo* GC-MS analysis performed in parallel similarly demonstrated increased V_TCA_ following DCA administration. However, DCA-stimulated V_PDH_ estimated from 90-s ALCAR enrichment was lower than that inferred from HP [1-^13^C]ALCAR. Importantly, HP MRS captures real-time labeling kinetics whereas GC-MS reports metabolite pool sizes and isotopologue enrichment at a single terminal time point; differences between these readouts are therefore expected, particularly for rapidly exchanging pools such as ALCAR. At 90 s, ALCAR M+2 enrichment closely matched alanine M+3 enrichment, suggesting that ALCAR enrichment rapidly approached precursor enrichment, whereas glutamate labeling remained substantially lower. Under these conditions, a single early time point may underestimate the rate of pyruvate carbon entry into ALCAR, especially in DCA-treated animals with increased pyruvate oxidation. In contrast, HP MRS monitors dynamic labeling over the acquisition window, capturing the rapid appearance of labeled ALCAR. Notably, skeletal muscle glutamate requires substantially longer time to approach isotopic steady state,^17^ and its 90-s enrichment therefore reflects an early phase of TCA cycle labeling. Together, these differences in labeling kinetics explain the differing estimates obtained by HP MRS and GC-MS.

Prior studies suggest that DCA acutely alters pyruvate metabolism by activating PDH activity, lowering lactate and alanine levels, and enhancing pyruvate oxidation,^18^^,^^19^^,^^20^ while not all studies observed a corresponding increase in OXPHOS.^21^ These studies were primarily based on systemic metabolite measurements, biochemical assays, or *ex vivo* analyses to infer changes in mitochondrial substrate utilization. In our study, we did not observe any differences in lactate production between DCA-treated and untreated groups with *in vivo* HP MRS or *ex vivo* GM-MS*,* consistent with previous HP studies in skeletal muscle.^10^^,^^11^ These previous studies used HP [1-^13^C]lactate^10^^,^^11^ and reported decreased alanine transaminase (ALT)-mediated conversion of pyruvate to alanine in skeletal muscle following DCA treatment. We were unable to reliably detect alanine with HP MRS in this study, primarily because the C2 carbon of alanine has a rapid *T*_*1*_ relaxation and its resonance is split by strong J-coupling with a one-bond proton. Our GC-MS study, however, revealed a statistically significant decrease in total alanine concentration following DCA treatment which may be reflective of decreased ALT flux. Although the focus of this study pertains to the interrogation of mitochondrial metabolism, it is reassuring that our observed trends in cytosolic metabolism are mostly concordant with previous studies.

Here, we demonstrate that *in vivo* MRS with HP [2-^13^C,3-^2^H_3_]pyruvate can simultaneously probe V_PDH_ and V_TCA_ in skeletal muscle. The rapid appearance of HP ALCAR may indicate that V_PDH_ is not limited by V_TCA_. There is, therefore, a need to probe these pathways independently to better understand exercise-induced metabolic remodeling in skeletal muscle. For example, HP [2-^13^C,3-^2^H_3_]pyruvate MRS can be readily integrated with ^1^H or ^31^P MRS methods to provide a more comprehensive view of metabolic regulation. A recent ^1^H/^31^P MRS study reported decreased ALCAR levels and slower phosphocreatine recovery in the skeletal muscle of obese individuals compared with lean controls,^22^ and these findings have been linked to reduced ALCAR availability and decreased metabolic flexibility.^6^ These observations suggest an active role for ALCAR in mediating acetyl-CoA’s availability and fate. Indeed, exercise reversed ALCAR efflux in skeletal muscle by increasing acetyl-CoA oxidation in the TCA cycle.^23^ On the other hand, reduced CAT activity and limited capacity for ALCAR generation is also linked to metabolic inflexibility and insulin resistance.^17^ Given its unique ability to simultaneously interrogate ALCAR production and TCA cycle flux, HP [2-^13^C,3-^2^H_3_]pyruvate provides a powerful tool for gaining mechanistic insight into skeletal muscle metabolic remodeling in response to exercise, obesity, diabetes, and other systemic metabolic disorders.

Beyond skeletal muscle, this approach may also be valuable for evaluating therapeutic interventions targeting mitochondrial metabolism. DCA has been investigated as a treatment for several mitochondrial disorders because activated PDH promotes oxidative glucose metabolism and reduces lactate accumulation.^18^^,^^24^^,^^25^ However, the metabolic consequences of increasing PDH activity may vary depending on the specific site of dysfunction within the respiratory chain or TCA cycle. The ability of HP [2-^13^C]pyruvate to simultaneously assess PDH activity and downstream TCA-cycle metabolism may be useful for characterizing metabolic responses to DCA and related therapies in disease-specific animal models. Notably, the differential responses of ALCAR and glutamate suggest sensitivity to pathway-specific metabolic alterations beyond PDH activation alone. Such metabolic readouts could help identify bottlenecks distal to PDH and improve understanding of how therapeutic interventions influence mitochondrial function *in vivo*.

### Limitations of the study

Several limitations were identified in this study which should be considered when interpreting the data. First, the GC-MS measurements were performed at a single time point 90 s after intravenous bolus injection of [U-^13^C_3_]pyruvate. Because precursor enrichment is expected to continuously change following bolus injection, isotopic steady state is unlikely to be reached within the experimental time window thereby limiting rigorous kinetic analysis. Despite these limitations, the GC-MS experiment was designed as a bolus-chase experiment to closely mimic the conditions of an HP MRS study, and the observed labeling patterns provide a snapshot of early tracer incorporation which can inform interpretation of HP MRS results. Second, the parameter denoted here as TCA-cycle activity (V_TCA_) should be interpreted as the rate of pyruvate incorporation into the TCA cycle rather than as a direct measurement of total TCA-cycle turnover. Because the TCA cycle can be supplied by multiple substrates, including fatty acids, ketone bodies, and amino acids, labeling arising from pyruvate oxidation represents only a fraction of total oxidative metabolism. Thus, changes in pyruvate-derived glutamate labeling may not fully reflect alterations in total mitochondrial substrate oxidation. Third, direct quantitative comparison between HP MRS and GC-MS measurements is inherently difficult because the two techniques probe metabolism over different temporal windows. HP measurements are constrained by polarization decay and therefore focus on early metabolic events following probe delivery, whereas tissue enrichments measured by GC-MS continue to evolve throughout the labeling period. Fourth, only adult male rats were evaluated, and potential sex-specific differences in skeletal muscle PDH activation and TCA cycle responses to DCA remain to be determined. Finally, despite the enhanced signal sensitivity and prolonged *T*_*1*_ provided by HP [2-^13^C,3-^2^H_3_]pyruvate, dynamic characterization of HP [5-^13^C]glutamate remained challenging because of its low signal-to-noise ratio. Future studies incorporating higher temporal resolution measurements and complementary isotope-tracing experiments will help further define the kinetics of acetyl-CoA buffering and TCA-cycle labeling *in vivo*.

In conclusion, this study demonstrates that HP [2-^13^C,3-^2^H_3_]pyruvate enables *in vivo* assessment of divergent fates of PDH derived acetyl-CoA in skeletal muscle and reveals a disproportionate increase in HP [1-^13^C]ALCAR relative to HP [5-^13^C]glutamate following pharmacologic activation of PDH. These findings are consistent with ALCAR functioning as a dynamic buffer that accommodates excess PDH-derived acetyl-CoA when oxidative demand is not proportionally increased. By differentiating ALCAR labeling from TCA cycle turnover, HP [2-^13^C,3-^2^H_3_]pyruvate provides a valuable tool for probing the regulation of mitochondrial metabolism *in vivo*. Considering the established safety profile of HP ^13^C-pyruvate and multiple on-going clinical translations,^4^^,^^26^ this approach has translational potential for studying exercise physiology and metabolic disease, where impaired metabolic flexibility and altered acetyl-CoA handling are key features.

## Resource availability

### Lead contact

Requests for further information and resources should be directed to and will be fulfilled by the lead contact, Jae Mo Park (jaemo.park@utsouthwestern.edu).

### Materials availability

This study did not generate new unique reagents.

### Data and code availability

• The original GC-MS data are available from https://data.mendeley.com/datasets/5gxk56sdrz/1.
• This paper does not report original code.
• Any additional information required to reanalyze the data reported in this paper is available from the lead contact upon request.

## Acknowledgments

We gratefully acknowledge funding support from the U.S. Army Medical Research Acquisition Activity (W81XWH2210485, W81XWH2210490, and HT94252510616), the National Institutes of Health of the United States (P30DK127984, R01NS107409, R01HL170039, S10OD028490, S10OD018468, S10RR029119, and T32EB028093), and the 10.13039/100004917Cancer Prevention & Research Institute of Texas (RP210099). Additionally, we acknowledge Duyen Nguyen of the Burgess Laboratory for her assistance with GC-MS sample preparation and data acquisition.

## Author contributions

S.C.B. and J.M.P. designed and supervised the study; S.H.L., Z.E., and S.W.D. performed the *in vivo* imaging and tissue collection; A.C., B.K., and X.F. performed the *ex vivo* isotopomer analysis; M.T.H. and Z.K. synthesized the isotope-labeled compounds; S.H.L., A.C., S.C.B., and J.M.P. drafted the manuscript. All authors contributed to manuscript editing.

## Declaration of interests

The authors declare no competing interests.

## STAR★Methods

### Key resources table

**Table:** 

REAGENT or RESOURCE	SOURCE	IDENTIFIER
**Chemicals, peptides, and recombinant proteins**		

[2-^13^C]pyruvic acid	Sigma-Aldrich	Cat#W6624
[U-^13^C_3_]pyruvate	Cambridge Isotope Laboratories	Cat#CLM-2440
Dichloroacetate	Sigma-Aldrich	Cat#347795

**Deposited data**		

GC-MS	This paper; Mendeley Data	https://doi.org/10.17632/5gxk56sdrz.1

**Experimental models: Organisms/strains**		

Rat/Sprague-Dawley	Charles River Laboratories	Cat#400SASSD

**Software and algorithms**		

GraphPad Prism version 10.4	GraphPad Software	https://www.graphpad.com/

### Experimental model and study participant details

#### Animals

Healthy male adult Sprague-Dawley rats (body weight: 451.7 ± 50.8 g; age: 10–12 weeks old) were included in this study. All animal experiments were conducted in compliance with institutional guidelines and approved by the UT Southwestern Institutional Animal Care and Use Committee (protocol#: 2016–101749). Every effort was made to minimize animal suffering, discomfort, and stress throughout the study. Animals were housed under controlled environmental conditions and monitored daily.

### Method details

#### Animal preparation and DCA treatment

Twelve rats were used in this study. Six rats were used for the *in vivo* MRS study, and the remaining six rats were used for the *ex vivo* GC-MS study. For DCA treatment, a 30 mg/mL solution of DCA in saline was prepared. Immediately following a rapid intravenous injection of 0.5 mL of the DCA solution, it was infused at a rate of 0.1 mL/3 min until the target dose of 100 mg/kg was achieved.^10^ Tissue collection or *in vivo* HP MRS was performed 45 min following the start of the DCA infusion.

#### Synthesis of sodium-[2-^13^C,3-^2^H_3_]Pyruvate and polarization

Sodium-[2-^13^C,3-^2^H_3_]pyruvate was synthesized from commercially available [2-^13^C]pyruvic acid using previously reported methods.^12^ For hyperpolarization, 190 μL of 2.8M sodium-[2-^13^C,3-^2^H_3_]pyruvate and 15 mM OX063 trityl radical in 3:2 (v/v) D_2_O:glycerol was loaded into the sample vial, and 17-mL of dissolution media (0.1 g/L ethylenediaminetetraacetic acid in D_2_O) was added into the dissolution syringe of a research fluid path (GE Healthcare, Waukesha, WI, USA). The fluid path was placed in a 5T SPINlab dynamic nuclear polarization (DNP) polarizer (GE Healthcare) and was polarized for 3–4 h at 0.8K. The HP substrate was subsequently dissolved in superheated dissolution media (130°C) to a final concentration of approximately 80 mM HP [2-^13^C,3-^2^H_3_]pyruvate (pH 7.5), which was immediately intravenously administered via a lateral tail vein catheter at a rate of 0.25 mL/s. The total administered dose of [2-^13^C,3-^2^H_3_]pyruvate was 1 mmol/kg body weight. In parallel, ∼0.5 mL of the remaining HP solution was analyzed in a 1 T ^13^C NMR spectrometer (Spinsolve, Magritek, Malvern, PA, USA) to estimate *in vitro* polarization levels, as previously described.^12^

#### MR protocol

All imaging was performed on a clinical 3T wide-bore MRI system (Discovery 750w, GE Healthcare) equipped with a custom-built ^13^C radiofrequency (RF) surface coil (single loop, diameter = 28 mm) placed over the right rectus femoris muscle. During each MR session, animals were continuously sedated with 1.5–3% isoflurane in oxygen (1.5 L/min) while maintaining the respiratory rate at 45–60 breaths/min. A ^1^H body coil was used for anatomic localization and B_0_ shimming. After shimming over the right biceps femoris region using a ^1^H PRESS sequence, time-resolved ^13^C free induction decay signals were acquired starting the beginning of the HP pyruvate bolus injection with a temporal resolution of 3 s. Each excitation was performed with a non-selective RF pulse (pulse width = 32 ms) with a nominal flip angle of 11.25°. Each animal received two injections of HP pyruvate: a baseline injection and a second injection 45 min following the initial DCA administration. The center frequency was set to 110 ppm, calculated from the water resonance at the right rectus femoris region,^27^ and the free induction decay signal was recorded every 0.1 ms (spectral width = 10 kHz, #spectral points = 4096). Detailed experimental setup for *in vivo* imaging is described in our previous study.^28^

#### Tissue extracts and GC-MS

A total of six healthy Sprague-Dawley rats were used for this study. Three rats were treated with DCA as described above, whereas the other three were untreated. To mimic the conditions of an HP MRS exam, a bolus of 80-mM [U-^13^C_3_]pyruvate was administered over 10–12 s (target dose of 1 mmol/kg, maximum administered bolus volume of 4 mL). Tissue samples from the bilateral hamstring muscles were collected and snap frozen in liquid nitrogen at approximately 90 s following the start of the pyruvate injection.

Frozen muscle tissue was extracted and derivatized for GC-MS analysis as follows. Approximately 20–30 mg of frozen muscle tissue was weighed and placed over ice. For enrichment measurements, the frozen tissue was mixed with 1 mL cold extraction solution which comprised of 8:8:3.9:0.1 (v/v/v/v) MeOH:acetonitrile:H_2_O:formic acid. For concentration measurements, the frozen tissue was mixed with 32 μL of an internal standard solution and 968 μL extraction solution. The mixture was homogenized over ice and subsequently incubated on ice for 10 min. Then, 75 μL 15% NH_4_HCO_3_ in H_2_O (w/v) was added and the mixture was incubated on ice for an additional 10 min. Next, the mixture was centrifuged (21,300g, 4 °C, 10 min) after which the supernatant was collected and dried over a stream of N_2_ gas at ambient temperature. The dried extract was reconstituted in 50 μL 1% methoxamine hydrochloride in pyridine (w/v) and incubated at 35 °C for 90 min. Next, 80 μL MTBSTFA with 1% TBDMCS (w/w) was added, and the mixture was incubated at 60 °C for 1 h. The solution was then cooled to room temperature prior to GC-MS analysis.

GC-MS analysis was performed using an Agilent 7890-A GC-MS system equipped with an HP-5ms column (30 m × 0.25 mm I.D., 0.25 mm film thickness; Agilent J&W) combined with an Agilent 5975-C mass spectrometer (70 eV, electron ionization source). A 1 μl injection volume for all samples was used, and the split mode was adjusted for optimal signal-to-noise ratio.

### Quantification and statistical analysis

Dynamic ^13^C MRS data were reconstructed using MATLAB (R2024b; MathWorks, Natick, MA, USA). The free-induction decay signal at each timepoint was apodized with a 5-Hz Gaussian filter, 2-fold zero-filling was applied, and a Fourier transform was performed, after which 0^th^ and 1^st^ order phase corrections were applied. Metabolites were quantified from time-accumulated spectra over 0–90 s by integrating the corresponding resonances in pure absorption mode. For comparison of baseline and DCA-treated spectra, metabolite signals were normalized to the total HP ^13^C signal (TC), which corresponds to the sum of the [2-^13^C]pyruvate, [2-^13^C]pyruvate-hydrate, [2-^13^C]lactate, [5-^13^C]glutamate, and [1-^13^C]ALCAR resonant peak areas. DCA-induced changes in metabolites were evaluated using paired t-tests (two-tailed, α = 0.05).

Quantification of GC-MS data was performed as follows: Fractional enrichment measurements were performed by calculating the ratio of individual ion peak areas to that of other isotopomers. For concentration measurements, individual ion peak areas were compared to that of an internal standard. Differences between control and DCA-treated rats were evaluated using unpaired t-tests (two-tailed, α = 0.05).

The statistical details of experiments can be found in the Results section. All statistical analyses were conducted using GraphPad Prism (version 10.4; GraphPad Software, San Diego, CA, USA). All data are presented as mean ± standard deviation. ∗, ∗∗, and ∗∗∗ indicate *p* < 0.05, *p* < 0.01, and *p* < 0.001, respectively.

## References

[1] Holloszy J.O. (1967). Biochemical adaptations in muscle. Effects of exercise on mitochondrial oxygen uptake and respiratory enzyme activity in skeletal muscle. J. Biol. Chem..

[2] Mogensen M., Sahlin K., Fernström M., Glintborg D., Vind B.F., Beck-Nielsen H., Højlund K. (2007). Mitochondrial respiration is decreased in skeletal muscle of patients with type 2 diabetes. Diabetes.

[3] Golman K., in 't Zandt R., Thaning M. (2006). Real-time metabolic imaging. Proc. Natl. Acad. Sci. USA.

[4] Park J.M., Harrison C.E., Ma J., Chen J., Ratnakar J., Zun Z., Liticker J., Reed G.D., Chhabra A., Haller R.G. (2021). Hyperpolarized (13)C MR Spectroscopy Depicts in Vivo Effect of Exercise on Pyruvate Metabolism in Human Skeletal Muscle. Radiology.

[5] Rahimi Y., Camporez J.P.G., Petersen M.C., Pesta D., Perry R.J., Jurczak M.J., Cline G.W., Shulman G.I. (2014). Genetic activation of pyruvate dehydrogenase alters oxidative substrate selection to induce skeletal muscle insulin resistance. Proc. Natl. Acad. Sci. USA.

[6] Lindeboom L., Nabuurs C.I., Hoeks J., Brouwers B., Phielix E., Kooi M.E., Hesselink M.K.C., Wildberger J.E., Stevens R.D., Koves T. (2014). Long-echo time MR spectroscopy for skeletal muscle acetylcarnitine detection. J. Clin. Investig..

[7] Schroeder M.A., Atherton H.J., Dodd M.S., Lee P., Cochlin L.E., Radda G.K., Clarke K., Tyler D.J. (2012). The cycling of acetyl-coenzyme A through acetylcarnitine buffers cardiac substrate supply: a hyperpolarized 13C magnetic resonance study. Circ., Cardiovasc. Imaging.

[8] Muoio D.M., Noland R.C., Kovalik J.P., Seiler S.E., Davies M.N., DeBalsi K.L., Ilkayeva O.R., Stevens R.D., Kheterpal I., Zhang J. (2012). Muscle-specific deletion of carnitine acetyltransferase compromises glucose tolerance and metabolic flexibility. Cell Metab..

[9] Josan S., Park J.M., Hurd R., Yen Y.F., Pfefferbaum A., Spielman D., Mayer D. (2013). In vivo investigation of cardiac metabolism in the rat using MRS of hyperpolarized [1-13C] and [2-13C]pyruvate. NMR Biomed..

[10] Park J.M., Josan S., Mayer D., Hurd R.E., Chung Y., Bendahan D., Spielman D.M., Jue T. (2015). Hyperpolarized 13C NMR observation of lactate kinetics in skeletal muscle. J. Exp. Biol..

[11] Park J.M., Josan S., Hurd R.E., Graham J., Havel P.J., Bendahan D., Mayer D., Chung Y., Spielman D.M., Jue T. (2021). Hyperpolarized NMR study of the impact of pyruvate dehydrogenase kinase inhibition on the pyruvate dehydrogenase and TCA flux in type 2 diabetic rat muscle. Pflügers Archiv.

[12] Huynh M.T., Erfani Z., Kovács Z., Park J.M. (2024). Hyperpolarized [2-(13)C, 3-(2)H(3)]Pyruvate Detects Hepatic Gluconeogenesis In Vivo. ACS Sens..

[13] Wang Z.J., Ohliger M.A., Larson P.E.Z., Gordon J.W., Bok R.A., Slater J., Villanueva-Meyer J.E., Hess C.P., Kurhanewicz J., Vigneron D.B. (2019). Hyperpolarized (13)C MRI: State of the Art and Future Directions. Radiology.

[14] Howlett R.A., Heigenhauser G.J., Hultman E., Hollidge-Horvat M.G., Spriet L.L. (1999). Effects of dichloroacetate infusion on human skeletal muscle metabolism at the onset of exercise. Am. J. Physiol..

[15] Stacpoole P.W. (1989). The pharmacology of dichloroacetate. Metabolism.

[16] Jucker B.M., Dufour S., Ren J., Cao X., Previs S.F., Underhill B., Cadman K.S., Shulman G.I. (2000). Assessment of mitochondrial energy coupling in vivo by 13C/31P NMR. Proc. Natl. Acad. Sci. USA.

[17] Seiler S.E., Martin O.J., Noland R.C., Slentz D.H., DeBalsi K.L., Ilkayeva O.R., An J., Newgard C.B., Koves T.R., Muoio D.M. (2014). Obesity and lipid stress inhibit carnitine acetyltransferase activity. J. Lipid Res..

[18] Stacpoole P.W., Nagaraja N.V., Hutson A.D. (2003). Efficacy of dichloroacetate as a lactate-lowering drug. J. Clin. Pharmacol..

[19] Ferrando A.A., Chinkes D.L., Wolf S.E., Matin S., Herndon D.N., Wolfe R.R. (1998). Acute dichloroacetate administration increases skeletal muscle free glutamine concentrations after burn injury. Ann. Surg..

[20] Murray K., Dickson A.J. (1997). Dichloroacetate inhibits glutamine oxidation by decreasing pyruvate availability for transamination. Metabolism.

[21] Timmons J.A., Constantin-Teodosiu D., Poucher S.M., Greenhaff P.L. (2004). Acetyl group availability influences phosphocreatine degradation even during intense muscle contraction. J. Physiol..

[22] Klepochová R., Niess F., Meyerspeer M., Slukova D., Just I., Trattnig S., Ukropec J., Ukropcová B., Kautzky-Willer A., Leutner M., Krššák M. (2024). Correlation between skeletal muscle acetylcarnitine and phosphocreatine metabolism during submaximal exercise and recovery: interleaved (1)H/(31)P MRS 7 T study. Sci. Rep..

[23] Seiler S.E., Koves T.R., Gooding J.R., Wong K.E., Stevens R.D., Ilkayeva O.R., Wittmann A.H., DeBalsi K.L., Davies M.N., Lindeboom L. (2015). Carnitine Acetyltransferase Mitigates Metabolic Inertia and Muscle Fatigue during Exercise. Cell Metab..

[24] Michelakis E.D., Webster L., Mackey J.R. (2008). Dichloroacetate (DCA) as a potential metabolic-targeting therapy for cancer. Br. J. Cancer.

[25] Zhang Y., Sun M., Zhao H., Wang Z., Shi Y., Dong J., Wang K., Wang X., Li X., Qi H., Zhao X. (2023). Neuroprotective Effects and Therapeutic Potential of Dichloroacetate: Targeting Metabolic Disorders in Nervous System Diseases. Int. J. Nanomed..

[26] Punwani S., Larson P.E.Z., Laustsen C., VanderMeulen J., Ardenkjær-Larsen J.H., Autry A.W., Bankson J.A., Bernard J., Bok R., Bertelsen L.B. (2025). Consensus recommendations for hyperpolarized [1-(13)C]pyruvate MRI multi-center human studies. Magn. Reson. Med..

[27] Ma J., Chen J., Reed G.D., Hackett E.P., Harrison C.E., Ratnakar J., Schulte R.F., Zaha V.G., Malloy C.R., Park J.M. (2021). Cardiac T2 ∗ measurement of hyperpolarized (13) C metabolites using metabolite-selective multi-echo spiral imaging. Magn. Reson. Med..

[28] Chen J., Hackett E.P., Kovacs Z., Malloy C.R., Park J.M. (2021). Assessment of hepatic pyruvate carboxylase activity using hyperpolarized [1-(13) C]-l-lactate. Magn. Reson. Med..

