# Supplementary material for: Differential activation of TCA cycle activity and PDH flux by dichloroacetate in skeletal muscle measured by hyperpolarized [2-13C,3-2H3]pyruvate
Source: iScience. 2026 Aug 27;29(9):117325. doi: 10.1016/j.isci.2026.117325 (PMC13546930; doi:10.1016/j.isci.2026.117325)
Supplement: Supplementary file 1 [file mmc1.pdf]

iScience, Volume *29*

Supplemental information

Differential activation of TCA cycle activity and PDH flux by dichloroacetate in skeletal muscle measured by hyperpolarized [2-^{13}C,3-$^{2}H_3$]pyruvate

Sung-Han Lin, Andrew Cho, Mai T. Huynh, Zohreh Erfani, Blanka Kucejova, Sasanka Wathukara Dewage, Thomas Jue, Xiaorong Fu, Zoltán Kovács, Shawn C. Burgess, and Jae Mo Park

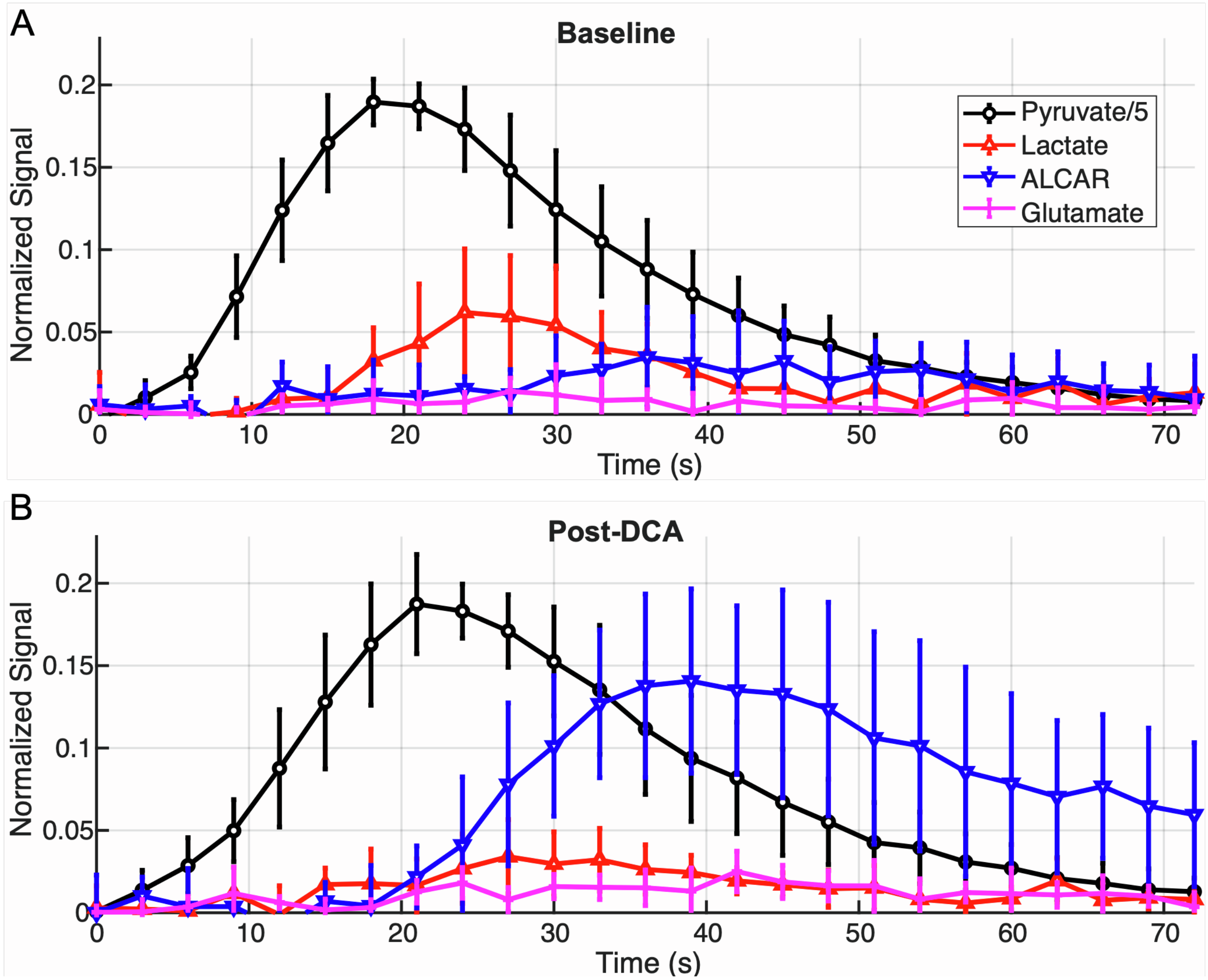

Figure S1. Time courses of HP [2-^{13}C]pyruvate and its products in skeletal muscle. For each rat, metabolite signals were normalized to the maximum [2-^{13}C]pyruvate peak. [2-^{13}C]pyruvate (black), [2-^{13}C]lactate (red), [1-^{13}C]ALCAR (blue), and [5-^{13}C]glutamate (magenta) in rat skeletal muscle at (A) baseline and (B) post-DCA. Error bars indicate the standard deviations across animals.

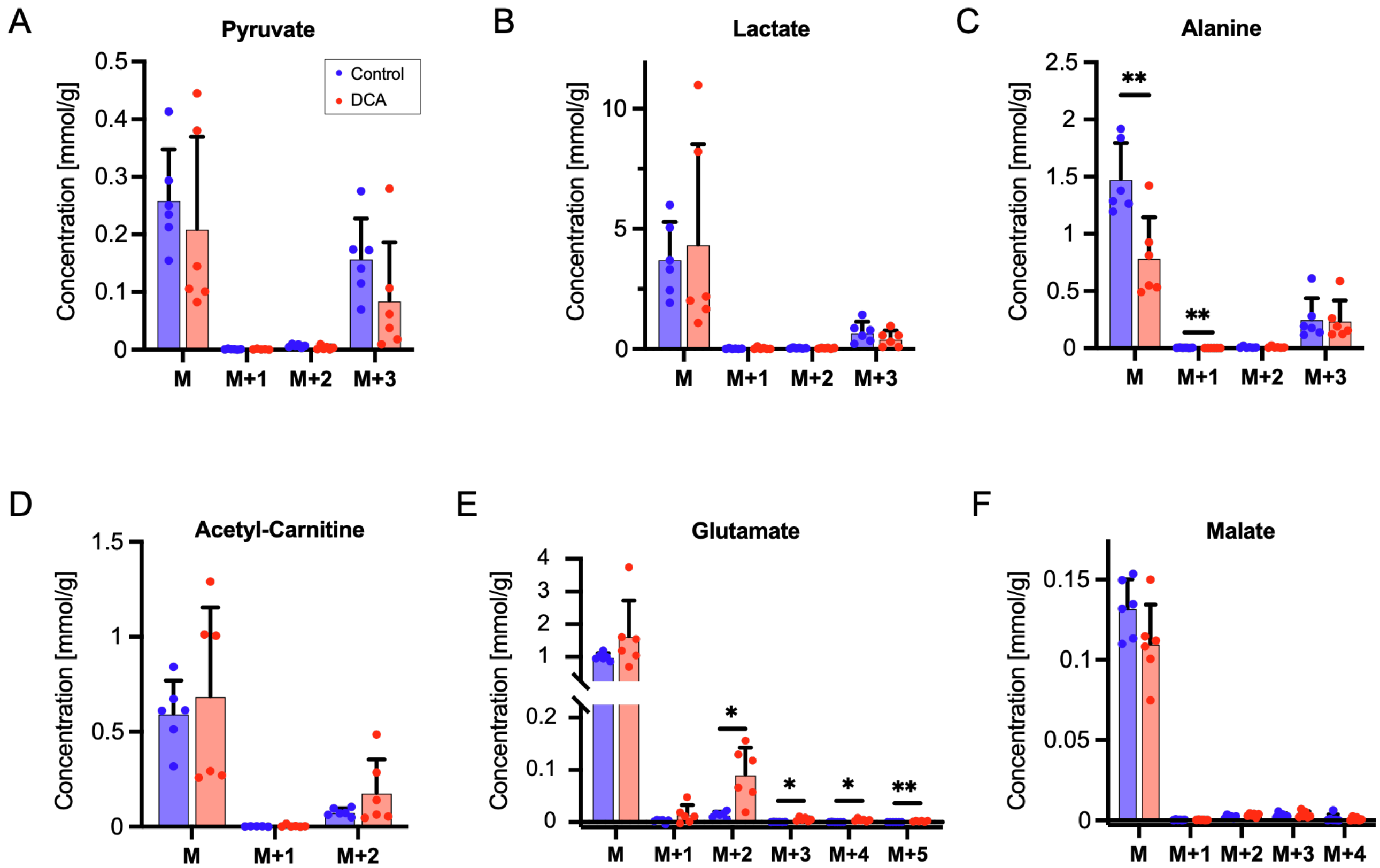

Figure S2. Isotopologue-resolved concentrations of key metabolites in skeletal muscle after bolus injection of [U-$^{13}C_3$]pyruvate. Isotopologue concentrations of (A) pyruvate, (B) lactate, (C) alanine, (D) acetyl-L-carnitine, (E) glutamate and (F) malate. Error bars indicate the standard deviations across animals. Data are represented as mean +/- SD. Unpaired t-tests (two-tailed, α = 0.05). ** *P* < 0.01.
